# Nonlinear response of Northern Hemisphere stratospheric polar vortex to the Indo–Pacific warm pool (IPWP) Niño

**DOI:** 10.1038/s41598-019-49449-7

**Published:** 2019-09-23

**Authors:** Xin Zhou, Quanliang Chen, Fei Xie, Jianping Li, Minggang Li, Ruiqiang Ding, Yanjie Li, Xin Xia, Zhigang Cheng

**Affiliations:** 1 Plateau Atmosphere and Environment Key Laboratory of Sichuan Province, College of Atmospheric Science, Chengdu University of Information Technology, Chengdu, China; 2 College of Global Change and Earth System Science, Beijing Normal University, Beijing, China; 3 Key Laboratory of Physical Oceanography–Institute for Advanced Ocean Studies, Ocean University of China and Qingdao National Laboratory for Marine Science and Technology, Qingdao, 266003 China; 4 State Key Laboratory of Numerical Modeling for Atmospheric Sciences and Geophysical Fluid Dynamics (LASG), Institute of Atmospheric Physics, Chinese Academy of Sciences, Beijing, China

**Keywords:** Atmospheric science, Climate and Earth system modelling, Atmospheric dynamics

## Abstract

Variations in tropical sea surface temperatures (SST) have pronounced impacts on the stratospheric polar vortex, with the role of El Niño being the focus of much research interest. However, the Indo–Pacific warm pool (IPWP), which is the warmest body of seawater in the world, has received less attention. The IPWP has been warming in recent years. This paper presents for the first time the remarkable nonlinearity in Northern Hemisphere (NH) stratospheric circulation and temperature response to IPWP warming (the so-called IPWP Niño) in boreal winter. The magnitude of NH stratospheric vortex weakening is strong and significant in case of moderate IPWP Niño, but is weak and insignificant in strong IPWP Niño case. This phenomenon is robust in both the historical simulations and observations. An idealized model experiments forced with linear varying SST forcing in the IPWP region isolate the nonlinearities arising from IPWP Niño strength. Westward extension of precipitation into the Maritime Continent drives attenuation and westward shift of extratropical waves during strong IPWP Niño events. Linear wave interference analysis reveals this leads to weak interference between the climatological and anomalous stationary waves and thereby a weak response of the stratospheric vortex. These findings imply a distinct stratospheric vortex response to the IPWP Niño, and provide extended implications for the surface climate in the NH.

## Introduction

Previous studies have demonstrated the importance of tropical sea surface temperatures (SST) in modulating the stratospheric polar vortex, and the El Niño–Southern Oscillation (ENSO) is one of the most discussed aspects of this relationship^1–7^. The tropospheric Rossby wave anomaly associated with ENSO warming, or El Niño, which projects onto the climatological stationary wave over the North Pacific, intensifies the vertical propagation of planetary scale Rossby waves upwards into the stratosphere in the Northern Hemisphere (NH) during the boreal winter and therefore leads to a markedly warmer and weaker polar vortex^4,8–12^. However, for the cold phase of ENSO, La Niña, reanalysis data reveal a weak and insignificant polar stratospheric cooling^11,13,14^, though a recent study has reported a robust strengthened and cooled vortex associated with La Niña^15^. Possible explanations for this asymmetric response to the opposite phases of ENSO include inherent differences in extratropical teleconnections^16–19^ and the background SST in the cold tongue area^20^.

Each El Niño event can differ in magnitude, which is commonly measured by the corresponding SST anomalies in the eastern tropical Pacific^21,22^. Recent projections indicate that very strong El Niño events, also called extreme or super El Niño events, are expected to increase in frequency in the future under global warming^23–27^. A major impact on stratospheric circulation has been observed during the three unusually strong El Niño events that have occurred during the satellite era; i.e., the 1982/83, 1997/98, and 2015/16 El Niño events^28^. Domeisen *et al*.^2^ reviewed on the question that whether strong El Niño events lead to a proportionately stronger stratospheric response compared with moderate El Niño events, where discrepancy was noted. Garfinkel *et al*.^29^ shown stronger response to extreme El Niño comparing with that to moderate El Niño in North Pacific Sea Level Pressure field with weak nonlinearity in the location of the extrema; Weinberger *et al*.^30^ suggested that stratospheric response to extreme El Niño is weaker than proportionate amplitude of El Niño strength. But some idealized experiments have different results. Zhou *et al*.^31^ found that the magnitude of the simulated stratospheric response to extreme El Niño events was four times stronger than that to moderate ones in late winter and early spring; Jimenez-Esteve and Domeisen^32^ showed that strong El Niño with double amplitude of its moderate counterpart yields more than twice the impacts of moderate El Niño in North Pacific atmosphere.

The SST anomalies in different tropical regions have different impacts on the stratosphere. The tropical Indian Ocean, for example, has become a research focus due to sustained warming in recent years^33^. When isolated from ENSO variability, the Indian Ocean warming induces increased precipitation over the Indian Ocean, drives teleconnections to the NH extratropics, and leads to positive northern annular mode (NAM) response in an opposite sign of El Niño impacts^34^. Thus it partially attenuate El Niño impacts on the stratospheric polar vortex during the El Niño winter^34,35^.

However, the IPWP, spanning the tropical western Pacific and eastern Indian oceans where the sea surface temperature (SST) exceeds 28 °C year-round^36^, being one of the major suppliers of atmospheric releasing heat and water vapor, leads to the most intense global air convection and climate influences globally^37^, yet has received less attention than ENSO in the regard of stratospheric impacts. Since the 1980s, the IPWP has experienced a continuous expansion, with significant increases in SST^38,39^. Similar to the definition of ENSO events^40^, anomalous warming/cooling of the IPWP (hereafter called IPWP Niño/Niña) is identified when 5-month running mean of the SST variations in the IPWP (TI_(IPWP)_) exceed half the standardized deviation (0.5σ)^41^. It is well appreciated that SST variations in the IPWP is correlated with ENSO through the “atmospheric bridge”^42^. El Niño-induced surface changes in surface heat fluxes can lead to warming in the IPWP^43^; On the other hand, the extension, displacements and intensity variations of the IPWP are known to affect the onset, intensity, and period of ENSO^44^. The IPWP Niño/Niña events can occur with or without ENSO events, each case accounting for about half episodes of IPWP Niño/Niña events from 1980 to 2015 according to Zhou *et al*.^41^. When isolated from ENSO variability, the IPWP can significantly influence the stratospheric circulation and temperature^41^ and is even more efficient in pumping water vapor from the troposphere into the stratosphere than ENSO^45^.

Enhanced convection excited by the IPWP Niño launches equatorial planetary waves in the form of Kelvin and equatorial Rossby wave responses^46^, influencing the tropopause temperature with smaller zonal asymmetries than that of ENSO and thereby leading more upward transport of water vapor into the stratosphere^45^. Temperature perturbations by adiabatic heating associated with IPWP Niño can radiate away into the mid-latitude troposphere, which bring about extratropical teleconnection^47^. During boreal winter, anomalous SST warming associated with IPWP Niño can excite NH extratropical teleconnections that project onto the positive phase of the Pacific–North America (PNA) pattern in mid–high latitudes, intensifying the upward propagation of planetary waves into the stratosphere and, in turn, warming and weakening the NH stratospheric vortex^41^. Note that the wave pattern excited by IPWP Niño is located further west than the PNA pattern, a fact that may be related to the different wave source locations from that of ENSO. Asymmetric response between the IPWP Niño and IPWP Niña–i.e. a same-signed response during opposite IPWP phase–has already been shown^41^, which is linked to asymmetries in extratropical IPWP teleconnections. However, it remains unclear whether a nonlinear relationship exist between the NH stratospheric response and IPWP Niño strength.

This study constitutes the first attempt to investigate the nonlinearities in the NH stratospheric polar vortex response to IPWP Niño in both observations and atmospheric model. We first show the relationship between the SST anomalies associated with IPWP Niño events and the polar stratospheric response in CMIP5 historical simulations, for identification of the nonlinearity and turning point of the stratospheric response. Using this turning point as a threshold, we confirm the nonlinearity by comparing the composite stratospheric circulation and temperature anomalies during strong and moderate IPWP Niño winters based on reanalysis datasets. Finally, we isolate the nonlinearities arising solely through changes in IPWP Niño strength by forcing a linear change in the amplitude of the SST forcing in time-slice model simulations.

## Results

**Figure 1:**
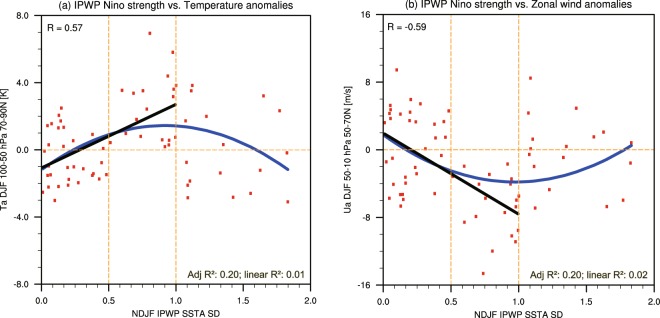
Scatterplots of the standardized NDJF SST anomalies (SSTA SD) of IPWP Niño events vs (**a**) the 70°N–90°N DJF zonal mean temperature anomalies at 100–50 hPa, and (**b**) the 50°N–70°N DJF zonal mean zonal wind anomalies at 50–10 hPa, based on CESM-WACCM4 historical simulations (1850–2005) provided by CMIP5. For all quantities, the variance linearly associated with ENSO and the QBO was regressed out before the data were stratified using the IPWP Niño strength. The correlation coefficient R for the linear fit (black; for SSTA SD from 0 to 1) is provided in the upper-left corner of each plot. A polynomial fit better describes the relationship, because adjusted R^2^ for a polynomial fit is larger than a linear fit (shown in the bottom-right corner of each plot).

To obtain a wide range of IPWP Niño events, we used CMIP5 outputs from a coupled climate system. Considering the good performance of the Community Earth System Model (CESM) Whole Atmosphere Community Climate Model, version 4 (WACCM4) (see Simulations for more information) in representing stratospheric variability, we analyzed simulations using CESM-WACCM4 from historical experiments covering the period 1850–2005 (see the Methods for details). Figure 1 shows scatterplots of the standardized NDJF TI_(IPWP)_, which indicates the strength of the IPWP Niño, versus the December–January–February (DJF) polar cap temperature anomalies between 70°N and 90°N and from 100 to 50 hPa (Fig. 1a), as well as the DJF zonal mean zonal wind anomalies averaged between 50°N and 70°N and 50 to 10 hPa (Fig. 1b). A clear nonlinearity in the stratospheric response is shown. That is, a parabolic fit of the polar cap temperature anomaly, which can be approximately expressed as *a* × *TI*_*(IPWP)*_^2^, better describes the relationship between IPWP Niño strength and the stratospheric response than does a linear fit. This is measured by the adjusted *R*^2^ (See the Method section), which is larger in case of polynomial fit (*R*^2^ ≈ 0.20 for both temperature and zonal wind) than in case of linear fit (*R*^2^ = 0.01 for temperature; *R*^2^ = 0.02 for zonal wind). Importantly, an inflection point occurs near the 1σ threshold. When IPWP Niño strength is below the 1σ threshold, the intensity of the polar stratospheric temperature and zonal wind anomalies increases with IPWP Niño strength. The correlation coefficient between IPWP Niño strength and the DJF polar vortex temperatures is *r* = 0.57, which suggests that the stronger the IPWP Niño event, the warmer the stratospheric anomaly. Correspondingly, the correlation coefficient for the zonal wind anomalies is *r* = −0.59; i.e., the stronger the IPWP Niño event, the weaker the polar vortex. In addition, the linear fit crossed zero close to, but lower than, the 0.5-SD threshold, supporting the use of 0.5σ to define IPWP Niño winters. However, when IPWP Niño strength exceeds the 1σ threshold, the response reverses so that it weakens with increasing IPWP Niño strength. An inflection point occurs near the 1σ threshold for both stratospheric temperature and circulation response.

Now we have seen the nonlinear stratospheric response in CESM-WACCM4 historical experiments. Note that this relationship is based on a single CMIP5 model (CESM-WACCM4), and can be different in other models, though not extended in this paper. However, two questions must be addressed here. Does the nonlinearity really exist? If so, does it come from IPWP Niño strength? Thus, we next investigate the change in stratospheric response with increasing IPWP Niño strength in the observations to validate the nonlinearity in the CMIP5 simulations, and then isolate the signal and tracing the nonlinearities from tropics to extratropical stratosphere based on idealized modeling results.

**Figure 2:**
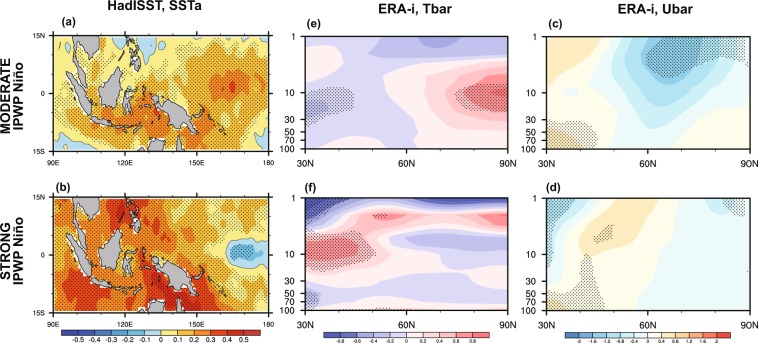
Composite of the (**a,b**) SST anomalies (°C) in the IPWP region (15°S–15°N, 90–180°E) from the HadISST (1979–2017), (**c,d**) zonal mean temperature anomalies (°C) and (**e,f**) zonal wind anomalies (m s^–1^) based on ERA-Interim (1979–2017), for (top) moderate IPWP Niño winters and (bottom) strong IPWP Niño winters. Before performing the composite analysis, the ENSO and QBO signals were removed from the wind and temperature fields. Eight Moderate IPWP Niño events and six strong IPWP Niño events listed in Table 1 are involved in the composite analysis. Stippling indicates anomalies that are significant at the 95% confidence level (Student’s *t*-test).

As the 1σ threshold has been shown to be the turning point of the stratospheric response (Fig. 1), we used this value to separate all of the IPWP Niño events into two groups: moderate and strong. That is, we defined a strong IPWP Niño as occurring when the winter mean TI_(IPWP)_ exceeds 1σ, with the moderate IPWP Niño group containing all of the remaining IPWP Niño events. The years included in each composite are listed in Table 1. The regional-mean composite SST anomaly during strong IPWP Niño events is obviously larger than that during moderate IPWP Niño events (Fig. 2a,b). We first present composite NH stratospheric temperature and circulation anomalies during moderate and strong IPWP Niño events, for the DJF average based on European Center for Medium Range Weather Forecasting (ECMWF) reanalysis dataset (ERA-Interim) (see Data for more information) for the period 1979–2017 (Fig. 2c–f). The general structure in the high latitudes during moderate IPWP Niño events resembles those reported by Zhou *et al*.^41^ (their Fig. 2) in terms of a significant stratospheric warming (peaking at about 1 K) together with a robust weakening of zonal mean zonal winds throughout the stratosphere. These significant stratospheric zonal mean temperature and wind anomalies indicate a weakening and warming stratospheric polar vortex. However, during strong IPWP Niño events the stratospheric temperature anomalies show a weak and insignificant cooling rather than a linearly stronger warming (Fig. 2d). Consistent with this, the polar stratospheric zonal winds are not weakened except in the vicinity of stratopause (Fig. 2f). Thus, observational evidence implies that a strong IPWP Niño has a weaker influence than its moderate counterpart.

**Table 1:** Moderate and strong IPWP Niño events in observations studied as composites.

Composite	Years
Moderate IPWP Niño	1987/88, 1995/96, 2002/03, 2004/05, 2006/07, 2008/09, 2009/10, 2013/14
Strong IPWP Niño	2001/02, 2003/04, 2012/13, 2014/15, 2015/16, 2016/17

**Figure 3:**
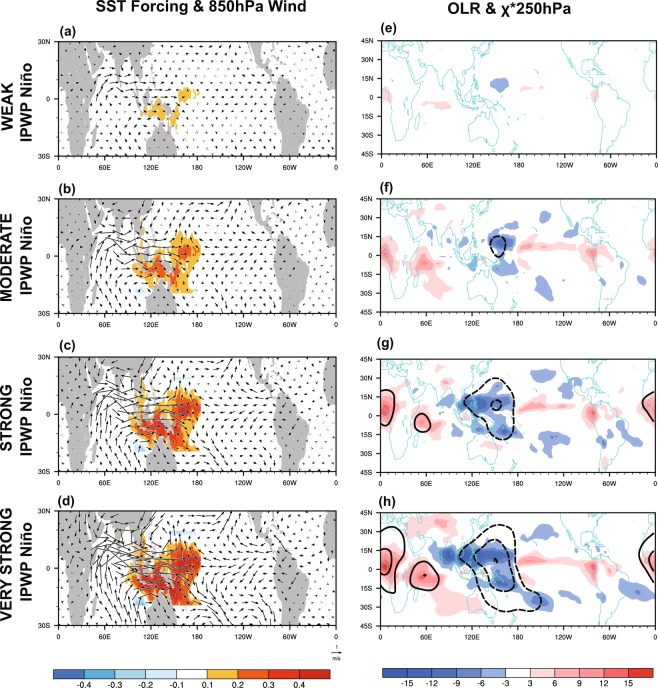
(**a–d**) The SST forcing (shading; units: °C) and 850-hPa winds (vectors; units: m s^−1^) response, and (**e–h**) outgoing longwave radiation response response (shading; W m^−2^) and 250-hPa eddy velocity potential response (contours; interval is 1.0 × 10^6^ m^2^ s^−2^) for (**a,e**) weak, (**b,f**) moderate, (**c,g**) strong, and (**d,h**) very strong IPWP Niño forcing.

To overcome the limited availability of observations and isolate the impact of IPWP Niño, we performed five 30-year sensitivity runs (R1–R5; see Simulations) using CESM-WACCM4 to mimic the IPWP Niño SST forcing with linear increasing strength, ranking as weak, moderate, strong, and very strong PWP Niño forcing (Fig. 3a–d) (see Simulations for more information). Following the experimental design by Jimenez-Esteve and Domeisen^32^, Cao *et al*.^48^, and Lin and Derome^49^, the nonlinearities arising solely through changes in IPWP Niño strength is isolated by forcing a linear change in the amplitude of the SSTs in the IPWP region and by forcing climatological SSTs elsewhere. It should be noted that the prescribed IPWP Niño SST forcing implicitly assumes that the SSTs in the IPWP region is applied entirely as a forcing, although it is likely that some fraction of the SST pattern is generated in response to atmospheric forcing, for example by a modulation of the Pacific Walker Circulation associated with ENSO^42^. We first compare the tropical response to the four linear IPWP Niño forcings. Here the magnitude of outgoing longwave radiation (OLR) response is used as a proxy for the intensity of tropical convection response. Low-level (850 hPa) convergence of zonal winds associated with Kelvin and Rossby waves towards the IPWP region coincides with upper level (250 hPa) divergence, leading to enhanced convection (negative OLR anomalies) over the IPWP region (Fig. 3e–h). This tropical circulation response is in agreement with previous findings from observations and ideal experiments with the linear baroclinic model^45^. Stronger IPWP Niño forcing induces stronger tropical precipitation anomalies, indicating larger adiabatic heating in the troposphere, acting as the Rossby wave source. Fletcher and Kushner^50^ using multiple configurations of atmospheric general circulation models also found an approximately proportional relationship between amplitude of zonally asymmetric components of tropical SST forcing and tropical precipitation response, accept in the Pacific cold tongue region due to thresholds for tropical convection. However, in cases of weak and moderate IPWP Niño precipitation anomalies centered over the equatorial from 150°E to 180°; in strong and very strong IPWP Niño cases, stronger ascent and larger precipitation penetrate deeper into the Maritime Continent.

**Figure 4:**
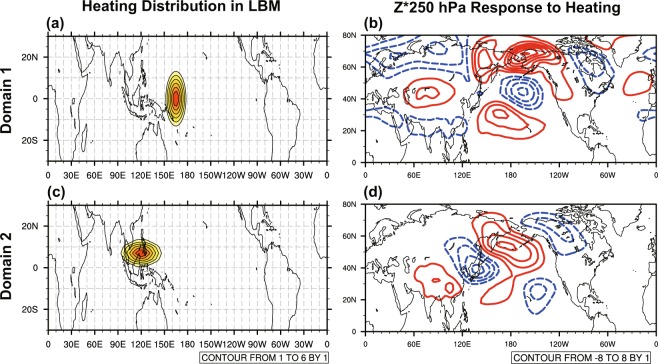
(left) Horizontal distribution of the idealized heating (K day^−1^) at the 0.45 sigma level used in the linear baroclinic model (LBM), and (right) the corresponding 250-hPa geopotential height response (gpm) for (**a,b**) domain 1 (150°E–180, 15°S–15°N) and (**c,d**) domain 2 (90°E–150°E, 0–15°N).

Many studies have found a large sensitivity of the extratropical response to the location and amplitude of the convective anomalies near the equator^49,51,52^. Goss and Feldstein^53^ applied a dynamical core of a climate model to run experiments with the heating field restricted to each of seven small domains located near or over the equator. They found that the heating anomalies over the equatorial Pacific from 150°E to 150°W force an anomalous low over the North Pacific and an anomalous high over the North America. However, heating anomalies over the Maritime Continent and Indian Ocean (50°E–150°E, 15°S–15°N) force opposite-signed extratropical response. Their findings suggest that the extratropical response in cases of strong and very strong IPWP Niño is very likely to have some cancellation between contribution from precipitation anomalies over the east part the IPWP region (150°E–180, 15°S–15°N; domain 1) and contribution from precipitation over the Maritime Continent (the west part of convection; 90°E–150°E, 0–15°N; domain 2). The essence of this argument involves the distinct extratropical response to convection over the two domains. In order to identify basic atmospheric processes associated with hearting in domain 1 and 2 separately, the linear baroclinic model (LBM) is used, with two heating fields restricted in domain 1 and 2 imposed separately in two runs (See Simulations for more information). Figure 4 shows the imposed heating fields and corresponding 300-hPa geopotential height response. The response to heating fields in domain 1 and 2 shows opposite-signed anomalies over the mid-latitude North Pacific and North America regions. The modeled results are in good agreement with findings in Goss and Feldstein^53^. The LBM solutions confirms the opposite-signed response between precipitation anomalies over the east and west part of the IPWP, which is likely to attenuate extratropical Rossby waves during strong IPWP Niño event.

**Figure 5:**
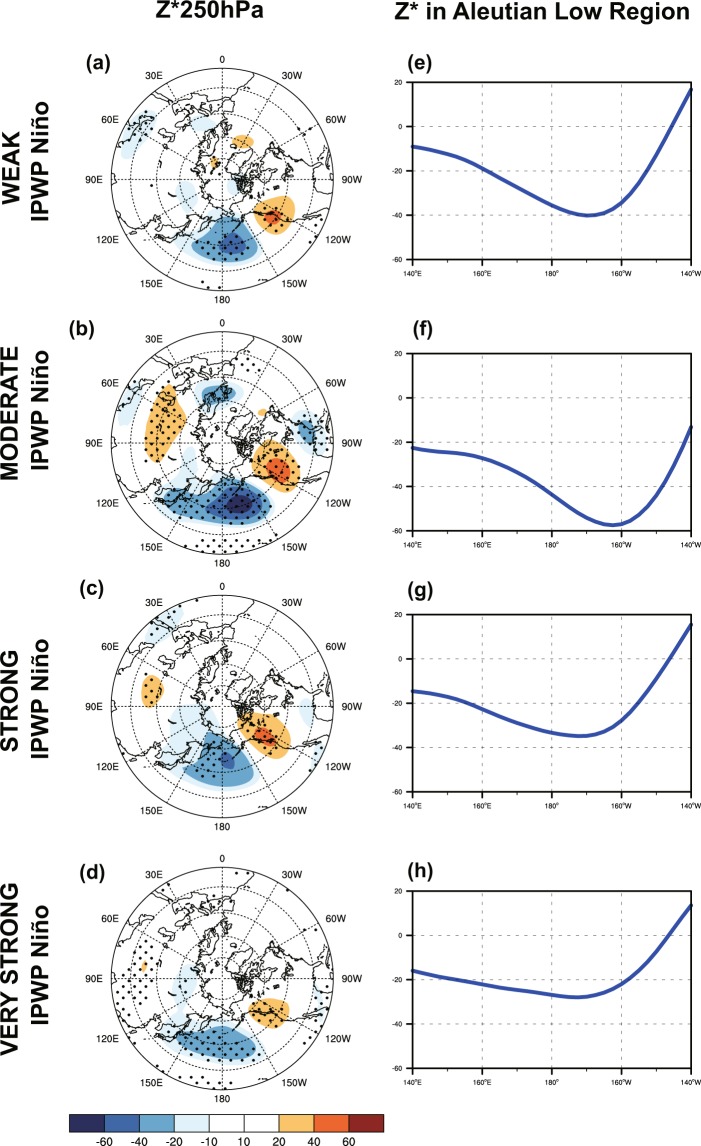
(**a–d**) 250-hPa wave geopotential height (Z * 250 hPa) anomalies, and (**e–h**) zonal variations of Z * 250 hPa in Aleutian low region for (**a,e**) weak, (**b,f**) moderate, (**c,g**) strong, and (**d,h**) very strong IPWP Niño (NDJ mean), based on WACCM4 simulations (R1–5). Stippling indicates anomalies that are significant at the 95% confidence level (Student’s *t*-test).

Thus, we next show the extratropical geopotential height response to the linear IPWP Niño forcing in Fig. 5. The four cases exhibit similar positive PNA-like wave trains in 250-hPa wave geopotential height field (Z^*^ 250 hPa; Z^*^ indicates that the zonal mean has been removed), with an anomalous deepened Aleutian low and an anomalous American high (Fig. 5). However, the strong and very strong IPWP Niño cases show a weaker negative anomaly in the Aleutian low region with a ~10° westward shift, comparing with weak and moderate cases. This is corresponding to westward extension of convection response in strong and very strong IPWP Niño cases. In addition, the weak amplitude of extratropical response suggests that contribution from the east part of IPWP Niño convection is largely cancelled out by contribution from the west part.

**Figure 6:**
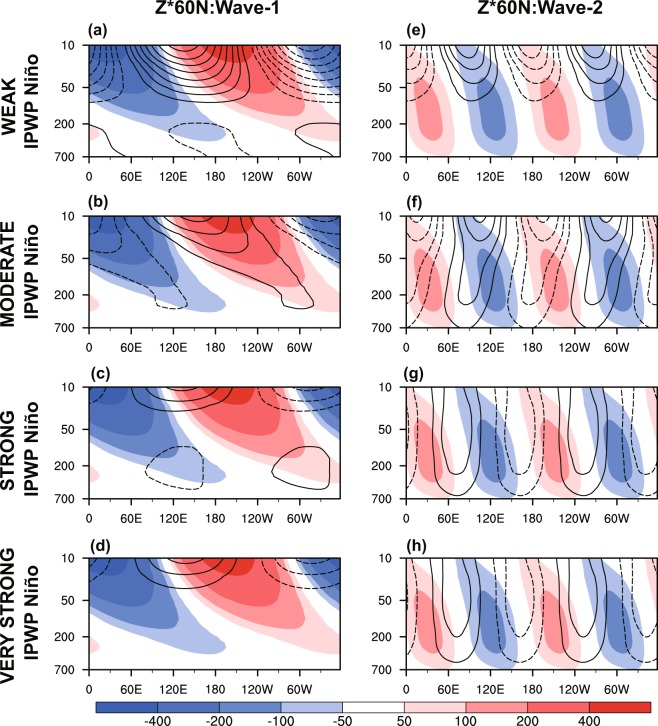
Latitude–height geopotential height associated with stationary waves of (**a–d**) wavenumber 1 and (**e–h**) wavenumber 2 at 60°N, for (**a,e**) weak, (**b,f**) moderate, (**c,g**) strong, and (**d,h**) very strong IPWP Niño (NDJ mean), based on WACCM4 simulations (R1–5). Contours show the wave response (contour interval is 5 m; negative contours are dashed) and shading shows the climatological stationary waves.

Previous studies have shown that when anomalous extratopical waves excited by tropical SST forcing propagate and dissipate in midlatitudes, the linear interference (phasing) of extratropical planetary waves determines the stratospheric vortex response^9,34,50,54–56^. Thus, we next examine the linear interference of extratropical planetary waves, with the wavenumber-1 and wavenumber-2 component playing the controlling role (Fig. 6). The phase difference between the anomalous wave geopotential height at 60°N (Z^*^ 60°N; Z^*^ indicates that the zonal mean has been removed) and its climatological mean is measured by the pattern correlation *r*_*zp*_ (See the Method for details). The entire depth of the troposphere and lower stratosphere is considered, in order to reveal a precursory planetary wave signal from the troposphere^57,58^^,^. The anomalous wave 1 projects weakly onto climatological wave 1 in weak IPWP Niño case (*r*_*zp*_ = 0.45), but projects strongly onto climatological wave 1 in moderate IPWP Niño case (*r*_*zp*_ = 0.78) (Fig. 6a,b); and the anomalous wave 2 is in quadrature with the climatological wave 2 (*r*_*zp*_ = −0.08 for weak IPWP Niño and *r*_*zp*_ = −0.09 for moderate IPWP Niño) (Fig. 6e,f). This pattern of positive wave-1 linear interference indicates increased wave activity flux entering the polar stratosphere, and is expected to weaken the polar vortex. However, for strong and very strong IPWP Niño the anomalous wave 1 is confined primarily to the stratosphere (Fig. 6c,d) and the anomalous wave project weakly onto the climatological wave for wave 2 (*r*_*zp*_ = 0.31 for strong IPWP Niño and *r*_*zp*_ = 0.29 for very strong IPWP Niño) (Fig. 5g,h). This wave pattern implies very weak wave activity flux into the stratosphere during strong and very strong IPWP Niño cases, and would corresponds to weakly disturbed polar vortex.

**Figure 7:**
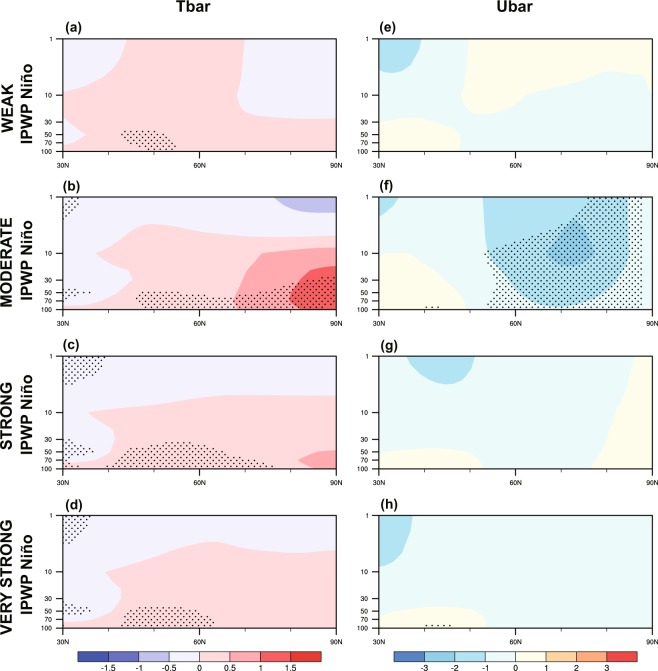
Latitude–pressure cross-sections of the DJF average of (**a–d**) zonal mean temperature (°C) and (**e–h**) zonal mean zonal wind (m s^-1^) response to (**a,e**) weak, (**b,f**) moderate, (**c,g**) strong and (**d,h**) very strong IPWP Niño, based on WACCM4 simulations (R1–5). Stippling indicates anomalies that are significant at the 95% confidence level (Student’s *t*-test).

Figure 7 presents the simulated stratospheric temperature and circulation response to the four linear IPWP Niño forcings. Consistent with anomalous strengthened wave activities, the moderate IPWP Niño leads to a significant warmer stratosphere at mid-to-high latitudes (Fig. 7b). However, the warming is weaker and insignificant in strong and very IPWP Niño cases, comparing with that in moderate case (Fig. 7c,d). Coherently, the zonal mean winds is markedly weakened during moderate IPWP Niño events (Fig. 7f), whereas the decrease during strong and very IPWP Niño events is not statistically significant (Fig. 7g,h). This characteristic of the stratospheric temperature and winds clearly validate the nonlinearity in the stratospheric response to IPWP Niño, which has been identified in CMIP5 simulations and observations above.

## Conclusions and Discussion

The combination of analyses using the historical relationship, reanalysis composites, and idealized experiments allows us to draw conclusion on the nonlinearity of IPWP Niño’s impacts on the NH stratospheric vortex. Anomalous warming SST associated with IPWP Niño launches precipitation over this area, which drives extratropical waves that further weaken the polar vortex. However, a nonlinear relationship between the amplitude of the IPWP Niño strength and the NH stratospheric vortex response is identified.

When the strength of the IPWP Niño is below the 1σ threshold, the intensity of the polar stratospheric temperature and zonal wind anomalies increases with increasing IPWP Niño strength. However, the response reverses and becomes weaker when IPWP Niño strength exceeds the 1σ threshold. This nonlinear relationship between IPWP SST anomalies and the NH stratospheric response is seen in CESM-WACCM4 CMIP5 historical simulations from 1850 to 2005. As 1σ is the point at which nonlinearity begins to develop in the stratospheric response, we used 1σ as the threshold to separate the IPWP Niño events into two groups (moderate and strong). By comparing the composition of the anomalous stratospheric temperature and circulations during moderate and strong IPWP Niño events, we found that the stratospheric circulation and temperature response is weaker during strong IPWP Niño events than during moderate events based on ERA-Interim for the period 1979–2017. That is, the stratospheric polar vortex is significantly warmer and weaker during moderate IPWP Niño events, whereas there is no significant signal during strong IPWP Niño events in the observations.

Idealized model experiments with scaled IPWP Niño SST forcing have been performed to isolate the signal from IPWP Niño and to validate the nonlinearities arising from IPWP Niño strength. After the westward extension of precipitation into the Maritime Continent, there is some cancellation between the extratropical response to the west part convection over the Maritime Continent and the response to convection over the east warm pool. This leading to the attenuation of extratropical wave response, with a ~10° westward shift in the Aleutian low region during strong IPWP Niño events. Thus, the wave-1 component moves from strong in phase with the climatological wave in moderate IPWP Niño case into rather weak in phase in strong IPWP Niño case and is mostly confined in upper stratosphere. This linear wave interference produces a significant vortex response in moderate IPWP Niño case but a very weak response in strong IPWP Niño case.

## Data and Methods

### Data

SST, temperature and circulation data are involved in observational composite analysis. For upper-air atmospheric fields, we use the monthly mean European Center for Medium Range Weather Forecasting (ECMWF) reanalysis dataset (ERA-Interim)^59,60^, which is extended from 1979 to near-real time, available at http://apps.ecmwf.int/datasets/. For SST, we use the monthly-mean product from the Hadley Centre Sea Ice and Sea Surface Temperature (HadISST) dataset^61^, available at http://www.metoffice.gov.uk/hadobs/hadisst/data/download.html.

The monthly output for the period 1850–2005 from historical simulations by CESM-WACCM4 in the CMIP5 archive (http://cmip-pcmdi.llnl.gov/cmip5/availity.html) allowed us to examine the stratospheric response to a wide range of IPWP Niño events. CESM-WACCM4 uses active ocean and sea ice components, and the model is forced using observed atmospheric composition changes from both natural (e.g., solar irradiance and volcanic aerosols) and anthropogenic (e.g., greenhouse gases, sulfate aerosols, and ozone) sources. The atmospheric component used was WACCM4. A representation of the QBO was achieved by relaxing the equatorial zonal wind between 86 and 4 hPa toward that observed^58^. To avoid the possible entanglement of QBO signals in our composite results from the fully coupled model, we regressed out the zonal wind at 50 hPa.

## Methods

We calculated the monthly anomalies by subtracting the long-term mean of each calendar month from each individual month. The linear trends were removed before analysis from the temperature, zonal wind, and geopotential height data using linear regression analysis. The quasi-biennial oscillation (QBO) index used was the standardized anomaly of equatorial 50-hPa zonal winds and it was used to regress out the QBO signal. The Niño 3.4 index was defined as the area mean SST anomaly over the region 5°S–5°N, 150°W–90°W (http://www.cpc.noaa.gov/data/indices/). All statistical tests were performed using the two-tailed Student’s *t*-test. To better represent the reversing manifestation of the nonlinearities, the composite results and model results are not scaled by IPWP Niño strength.

To diagnose the linear interference of Rossby waves, we used the pressure-weighted correlation *r*_*zp*_ between the anomalous wave geopotential height at 60 N (Z^*^ 60°N; Z^*^ indicates that the zonal mean has been removed) and its climatological mean, following the framework of Fletcher and Cassou^34^. The weights are based on the relative thickness of each of the vertical layers from 700 to 10 hPa. Since the The correlation is computed separately for the zonal wavenumber 1 and 2 components of Z * 60°N, which are filtered using a Fourier transform.

The adjusted *R*^2^ (Eq. 3.30 of Chatterjee and Hadi^62^) is used to quantify the added value in using a polynomial best fit (e.g., T ~ *a* × *TI*_*(IPWP)*_^2^) instead of a linear best fit (e.g., T ~ *b* × *TI*_*(IPWP)*_). The adjusted *R*^2^ takes into account the likehood that a polynomial predictor will reduce the residuals by unphysically over-fitting the data. The polynomial fit could be preferred if the adjusted *R*^2^ for the polynomial fit is larger as compared to the linear *R*^2^.

### Simulations

The time-slice simulations were performed using CESM-WACCM4. The CESM-WACCM4, developed by the National Center for Atmospheric Research (NCAR), has 66 vertical levels extending from the ground to 4.5 × 10^−6^ hPa (~145 km geometric altitude), with vertical resolution of 1.1–1.4 km in both the TTL and the lower stratosphere (<30 km). It is unable to internally simulate the QBO signals but is forced using a 28-month fixed cycle (nudged QBO). The time-slice simulations presented in this paper were performed at a resolution of 1.9° × 2.5°, with interactive chemistry.

Following previous modeling work by Jimenez-Esteve and Domeisen^32^, Cao *et al*.^48^, and Lin and Derome^49^, the performed experiments mimic a linear IPWP Niño forcing. Five 30-yr time-slice experiments are conducted, consisting of a climatological run (R1) and four experiments (R2–R5) with IPWP Niño SST forcing. The simulations use prescribed fixed SSTs following the 1958–2016 monthly SST climatology. The winter mean composite SST anomalous pattern for moderate IPWP Niño (Fig. 2a) is multiplied by factors of 0.5, 1.0, 1.5, and 2.0 to generate weak (R2), moderate (R3), strong (R4) and very strong (R5) IPWP Niño forcing, respectively. The anomalous SST pattern is imposed in the IPWP region (15°S–15°N, 90–160°E), and SST forcing is set to zero outside of the IPWP region. To prevent discontinuities in SST forcing on the IPWP boundary, SST anomalies on the boundary are added to the five model grids centered at the IPWP boundary, with respective weights of 1.0, 0.75, 0.50, 0.25, and 0.0 from the inside to the outside model grids of the IPWP boundary. The experiments are each run for 33 years, removing the first 3 years as spin-up. The key point is that these model integrations isolate possible nonlinearities arising solely through changes in IPWP Niño strength.

The linear baroclinic model (LBM) is used as a diagnostic tool for studying the extratropical atmospheric response to idealized forcing^46,63–65^. It is constructed by linearizing the primitive equations about a 3D climatological basic state, with a T42 horizontal spectral resolution and 20 vertical levels on a sigma coordinate. The LBM is fully described in Watanabe and Kimoto^66^ and Watanabe and Jin^67^. In this paper, the heating fields restricted in domain 1 and 2 are imposed on boreal winter mean climatology derived from NCEP-NCAR reanalysis, with elliptical cosine-squared horizontal distribution shown in Fig. 3 and gamma vertical profile peaking at 400 hPa^64,68^. The LBM solutions provide evidence for the cancellation effect between the two domains in a linear framework.

## References

[1] Calvo N, Garcia-Herrera R, Garcia RR (2008). The ENSO Signal in the Stratosphere. Trends and Directions in. Climate Research.

[2] Domeisen DIV, Garfinkel CI, Butler AH (2019). The Teleconnection of El Nino Southern Oscillation to the Stratosphere. Rev. Geophys..

[3] Hamilton K (1993). An Examination of Observed Southern Oscillation Effects in the Northern-Hemisphere Stratosphere. J. Atmos. Sci..

[4] Hamilton K (1993). A general circulation model simulation of El Nino effects in the extratropical northern hemisphere stratosphere. Geophys. Res. Lett..

[5] Manzini E (2009). Atmospheric science: ENSO and the stratosphere. Nat. Geosci..

[6] Van Loon H, Labitzke K (1987). The Southern Oscillation .5. The Anomalies in the Lower Stratosphere of the Northern-Hemisphere in Winter and a Comparison with the Quasi-Biennial Oscillation. Mon. Weather Rev..

[7] Yulaeva E, Wallace JM (1994). The signature of ENSO in global temperature and precipitation fields derived from the microwave sounding unit. J. Clim..

[8] Garcia-Herrera, R., Calvo, N., Garcia, R. R. & Giorgetta, M. A. Propagation of ENSO temperature signals into the middle atmosphere: A comparison of two general circulation models and ERA-40 reanalysis data. *J. Geophys. Res*. **111**, 10.1029/2005JD006061 (2006).

[9] Garfinkel, C. I. & Hartmann, D. L. Different ENSO teleconnections and their effects on the stratospheric polar vortex. *J. Geophys. Res*. **113**, 10.1029/2008jd009920 (2008).

[10] Ineson S, Scaife AA (2009). The role of the stratosphere in the European climate response to El Nino. Nat. Geosci..

[11] Manzini E, Giorgetta MA, Esch M, Kornblueh L, Roeckner E (2006). The influence of sea surface temperatures on the northern winter stratosphere: Ensemble simulations with the MAECHAM5 model. J. Clim..

[12] Xie F, Li J, Tian W, Feng J, Huo Y (2012). Signals of El Niño Modoki in the tropical tropopause layer and stratosphere. Atmos. Chem. Phys..

[13] Free, M. & Seidel, D. J. Observed El Niño-Southern Oscillation temperature signal in the stratosphere. *J. Geophys. Res*. **114**, 10.1029/2009jd012420 (2009).

[14] Mitchell DM, Gray LJ, Charlton-Perez AJ (2011). The structure and evolution of the stratospheric vortex in response to natural forcings. J. Geophys. Res..

[15] Iza M, Calvo N, Manzini E (2016). The Stratospheric Pathway of La Nina. J. Clim..

[16] Butler, A. H. & Polvani, L. M. El Nino, La Nina, and stratospheric sudden warmings: A reevaluation in light of the observational record. *Geophys. Res. Lett*. **38**, 10.1029/2011GL04804 (2011).

[17] Domeisen DIV (2015). Seasonal Predictability over Europe Arising from El Nino and Stratospheric Variability in the MPI-ESM Seasonal Prediction System. J. Clim..

[18] Garfinkel CI, Hurwitz MM, Waugh DW, Butler AH (2013). Are the teleconnections of Central Pacific and Eastern Pacific El Nio distinct in boreal wintertime?. Clim. Dyn..

[19] Hoerling MP, Kumar A, Zhong M (1997). El Niño, La Niña, and the nonlinearity of their teleconnections. J. Clim..

[20] Fei X (2018). The key role of background sea surface temperature over the cold tongue in asymmetric responses of the Arctic stratosphere to El Niño–Southern Oscillation. Environ. Res. Lett..

[21] Capotondi A (2015). Understanding ENSO Diversity. Bull. Am. Meteorol. Soc..

[22] Timmermann A (2018). El Nino-Southern Oscillation complexity. Nature.

[23] Cai WJ (2014). Increasing frequency of extreme El Nino events due to greenhouse warming. Nature. Clim. Change.

[24] Cai WJ (2018). Increased variability of eastern Pacific El Nino under greenhouse warming. Nature.

[25] Latif M, Keenlyside NS (2009). El Nino/Southern Oscillation response to global warming. PNAS.

[26] Latif M, Semenov VA, Park W (2015). Super El Niños in response to global warming in a climate model. Clim. Change.

[27] Wang G (2017). Continued increase of extreme El Nino frequency long after 1.5 °C warming stabilization. Nature. Clim. Change.

[28] Rao J, Ren RC (2017). Parallel comparison of the 1982/83, 1997/98 and 2015/16 super El Nios and their effects on the extratropical stratosphere. Adv. Atmos. Sci..

[29] Garfinkel CI (2019). The salience of nonlinearities in the boreal winter response to ENSO: North Pacific and North America. Clim. Dyn..

[30] Weinberger, I., Garfinkel, C. I., White, I. P. & Oman, L. D. The salience of nonlinearities in the boreal winter response to ENSO: Arctic stratosphere and Europe. *Clim. Dyn*., 10.1007/s00382-019-04805-1 (2019). 10.1007/s00382-019-04805-1 PMC6769094 31631950

[31] Zhou X (2018). Does Extreme El Nino Have a Different Effect on the Stratosphere in Boreal Winter Than Its Moderate Counterpart?. J. Geophys. Res..

[32] Jimenez-Esteve B, Domeisen DIV (2019). Nonlinearity in the North Pacific Atmospheric Response to a Linear ENSO Forcing. Geophys. Res. Lett..

[33] Knutson TR, Delworth TL, Dixon KW, Stouffer RJ (1999). Model assessment of regional surface temperature trends (1949-1997). J. Geophys. Res..

[34] Fletcher CG, Cassou C (2015). The Dynamical Influence of Separate Teleconnections from the Pacific and Indian Oceans on the Northern Annular Mode. J. Clim..

[35] Rao Jian, Ren Rongcai (2015). A decomposition of ENSO’s impacts on the northern winter stratosphere: competing effect of SST forcing in the tropical Indian Ocean. Climate Dynamics.

[36] Yan XH, Ho CR, Zheng Q, Klemas V (1992). Temperature and size variabilities of the western Pacific Warm Pool. Science.

[37] Li JP (2013). Progress in air-land-sea interactions in Asia and their role in global and Asian climate change (in Chinese). Chinese. J Atmos Sci.

[38] Cravatte S, Delcroix T, Zhang DX, McPhaden M, Leloup J (2009). Observed freshening and warming of the western Pacific Warm Pool. Clim. Dyn..

[39] Graham NE, Barnett TP (1987). Sea Surface Temperature, Surface Wind Divergence, and Convection over Tropical Oceans. Science.

[40] Trenberth KE (1997). The definition of El Niño. Bull. Am. Meteorol. Soc..

[41] Zhou Xin, Li Jianping, Xie Fei, Ding Ruiqiang, Li Yanjie, Zhao Sen, Zhang Jiankai, Li Yang (2017). The effects of the Indo-Pacific warm pool on the stratosphere. Climate Dynamics.

[42] Liu, Z. Y. & Alexander, M. Atmospheric bridge, oceanic tunnel, and global climatic teleconnections. *Rev. Geophys*. **45**, 10.1029/2005RG000172 (2007).

[43] Stohl, A. *et al*. Stratosphere-troposphere exchange: A review, and what we have learned from STACCATO. *J. Geophys. Res*. **108**, 10.1029/2002JD002490 (2003).

[44] Picaut J, Ioualalen M, Menkes C, Delcroix T, McPhaden MJ (1996). Mechanism of the Zonal Displacements of the Pacific Warm Pool: Implications for ENSO. Science.

[45] Xie F (2018). Effect of the Indo-Pacific Warm Pool on Lower-Stratospheric Water Vapor and Comparison with the Effect of ENSO. J. Clim..

[46] Gill AE (1980). Some Simple Solutions for Heat-Induced Tropical Circulation. Q. J. R. Meteorolog. Soc..

[47] Hoskins BJ, Karoly DJ (1981). The steady linear response of a spherical atmosphere to thermal and orographic forcing. J. Atmos. Sci..

[48] Cao D (2019). Linear and nonlinear winter atmospheric responses to extreme phases of low frequency Pacific sea surface temperature variability. Clim. Dyn..

[49] Lin H, Derome J (2004). Nonlinearity of the Extratropical Response to Tropical Forcing. J. Clim..

[50] Fletcher CG, Kushner PJ (2013). Linear interference and the Northern Annular Mode response to tropical SST forcing: Sensitivity to model configuration. J. Geophys. Res..

[51] Geisler JE, Blackmon ML, Bates GT, Muñoz S (1985). Sensitivity of January Climate Response to the Magnitude and Position of Equatorial Pacific Sea Surface Temperature Anomalies. J. Atmos. Sci..

[52] Kiladis GN, Weickmann KM (1992). Circulation Anomalies Associated with Tropical Convection during Northern Winter. Mon. Weather Rev..

[53] Goss M, Feldstein SB (2017). Why Do Similar Patterns of Tropical Convection Yield Extratropical Circulation Anomalies of Opposite Sign?. J. Atmos. Sci..

[54] Fletcher CG, Kushner PJ (2011). The Role of Linear Interference in the Annular Mode Response to Tropical SST Forcing. J. Clim..

[55] Nishii K, Nakamura H, Orsolini YJ (2011). Geographical Dependence Observed in Blocking High Influence on the Stratospheric Variability through Enhancement and Suppression of Upward Planetary-Wave Propagation. J. Clim..

[56] Smith KL, Fletcher CG, Kushner PJ (2010). The Role of Linear Interference in the Annular Mode Response to Extratropical Surface Forcing. J. Clim..

[57] Birner T, Albers JR (2017). Sudden Stratospheric Warmings and Anomalous Upward Wave Activity Flux. Sola.

[58] Martius, O., Polvani, L. M. & Davies, H. C. Blocking precursors to stratospheric sudden warming events. *Geophys. Res. Lett*. **36**, 10.1029/2009GL038776 (2009).

[59] Simmons A, Uppala S, Dee D, Kobayashi S (2007). ERA-Interim: New ECMWF reanalysis products from 1989 onwards. ECMWF newsletter.

[60] Uppala S, Dee D, Kobayashi S, Berrisford P, Simmons A (2008). Towards a climate data assimilation system: Status update of ERA-Interim. ECMWF newsletter.

[61] Rayner NA (2003). Global analyses of sea surface temperature, sea ice, and night marine air temperature since the late nineteenth century. J. Geophys. Res..

[62] Chatterjee S, Ali SH (2013). Regression Analysis by Example, Fifth Edition. International Statistical Review.

[63] Matsuno T (1966). Quasi-geostrophic motions in the equatorial area. Journal of the Meteorological Society of Japan. Ser. II.

[64] Rodwell MJ, Hoskins BJ (1996). Monsoons and the dynamics of deserts. Q. J. R. Meteorolog. Soc..

[65] Webster PJ (1972). Response of the Tropical Atmosphere to Local, Steady Forcing. Mon. Weather Rev..

[66] Watanabe M, Kimoto M (2000). Atmosphere-ocean thermal coupling in the North Atlantic: A positive feedback. Q. J. R. Meteorolog. Soc..

[67] Watanabe M, Jin F-F (2003). A Moist Linear Baroclinic Model: Coupled Dynamical–Convective Response to El Niño. J. Clim..

[68] Matthews AJ, Hoskins BJ, Masutani M (2004). The global response to tropical heating in the Madden-Julian oscillation during the northern winter. Q. J. R. Meteorolog. Soc..

